# Application of survival tree analysis for exploration of potential interactions between predictors of incident chronic kidney disease: a 15-year follow-up study

**DOI:** 10.1186/s12967-017-1346-x

**Published:** 2017-11-28

**Authors:** Azra Ramezankhani, Maryam Tohidi, Fereidoun Azizi, Farzad Hadaegh

**Affiliations:** 1 Prevention of Metabolic Disorders Research Center, Research Institute for Endocrine Sciences, Shahid Beheshti University of Medical Sciences, Floor 3th, Number 24, Yemen Street, Shahid Chamran Highway, P.O. Box: 19395-4763, Tehran, Iran; 2 Endocrine Research Center, Research Institute for Endocrine Sciences, Shahid Beheshti University of Medical Sciences, Tehran, Iran

**Keywords:** Chronic kidney disease, Risk factors, Epidemiology, Survival tree analysis, Interaction

## Abstract

**Background:**

Chronic kidney disease (CKD) is a growing public health challenges worldwide. Various studies have investigated risk factors of incident CKD; however, a very few studies examined interaction between these risk factors. In an attempt to clarify the potential interactions between risk factors of CKD, we performed survival tree analysis.

**Methods:**

A total of 8238 participants (46.1% men) aged > 20 years without CKD at baseline [(1999–2001) and (2002–2005)], were followed until 2014. The first occurrence of CKD, defined as the estimated glomerular filtration rate (eGFR) < 60 ml/min/1.73 m^2^, was set as the main outcome. Multivariable Cox proportional hazard (Cox PH) regression was used to identify significant independent predictors of CKD; moreover, survival tree analysis was performed to gain further insight into the potential interactions between predictors.

**Results:**

The crude incidence rates of CKD were 20.2 and 35.2 per 1000 person-years in men and women, respectively. The Cox PH identified the main effect of significant predictors of CKD incidence in men and women. In addition, using a limited number of predictors, survival trees identified 12 and 10 subgroups among men and women, respectively, with different survival probability. Accordingly, a group of men with eGFR > 74 ml/min/1.73 m^2^, age ≤ 46 years, low level of physical activity, waist circumference ≤ 100 cm and FPG ≤ 4.7 mmol/l had the lowest risk of CKD incidence; while men with eGFR ≤ 63.4 ml/min/1.73 m^2^, age > 50 years had the highest risk for CKD compared to men in the lowest risk group [hazard ratio (HR), 70.68 (34.57–144.52)]. Also, a group of women aged ≤ 45 years and eGFR > 83.5 ml/min/1.73 m^2^ had the lowest risk; while women with age > 48 years and eGFR ≤ 69 ml/min/1.73 m^2^ had the highest risk compared to low risk group [HR 27.25 (19.88–37.34)].

**Conclusion:**

In this post hoc analysis, we found the independent predictors of CKD using Cox PH; furthermore, by applying survival tree analysis we identified several numbers of homogeneous subgroups with different risk for incidence of CKD. Our study suggests that two methods can be used simultaneously to provide new insights for intervention programs and improve clinical decision making.

## Background

Chronic kidney disease (CKD) is a growing public health challenges worldwide [1]. CKD is a major cause of not only progression to renal failure, but also excess cardiovascular morbidity and mortality. It affects 10–15% of the adult population around the world [1, 2]. In recent years, a silent CKD epidemic has been proposed by many authors [3, 4]. The outbreak is because of the inclusion of a large proportion of the elderly population within stage 3 CKD [5]. Also, in the developing countries, up to 40% of those identified with CKD in screening programs were often young [4]. Because early interventions could prevent or postpone the progression to end stage renal disease (ESRD), identification of risk factors is a major step in dealing with the CKD epidemic [6, 7].

The primary method for identifying predictors of incident CKD has been use of traditional regression models such as logistic or Cox proportional hazards (Cox PH) model. However, assessment of interactions in the presence of a large number of variables leads to a complicated model that can be difficult to fit and interpret [8–10]. For example, if we have 20 covariates, then there are (20 × 19)/2 = 190 two-factor interaction terms for including into regression models. On the other hand, if many interactions are examined and only the strongest included in the regression model, this would contribute to biased estimation of the effects and overly optimistic performance estimates [11]. Hence, in prediction of CKD, many studies have examined the interactions between only important risk factors such as hypertension or diabetes with other factors [12, 13].

Recursive partitioning or ‘decision trees’ are another class of nonparametric regressions which are widely used in many fields [14–17]. Survival trees are popular nonparametric alternatives to the Cox PH model, which have great flexibility and can automatically detect certain types of interactions. Moreover, they can naturally group subjects according to their length of survival based on their covariates patterns [11, 14, 18, 19]. The aims of the present study, conducted among Iranian adult population, were to investigate (1) the independent predictors of incident CKD separately among men and women using traditional statistical method and (2) the potential interactions between predictors to identify different risk groups for CKD incidence using survival tree analysis. We used data from the Tehran Lipid and Glucose Study (TLGS) for our investigation.

## Methods

### Study population

**Fig. 1:**
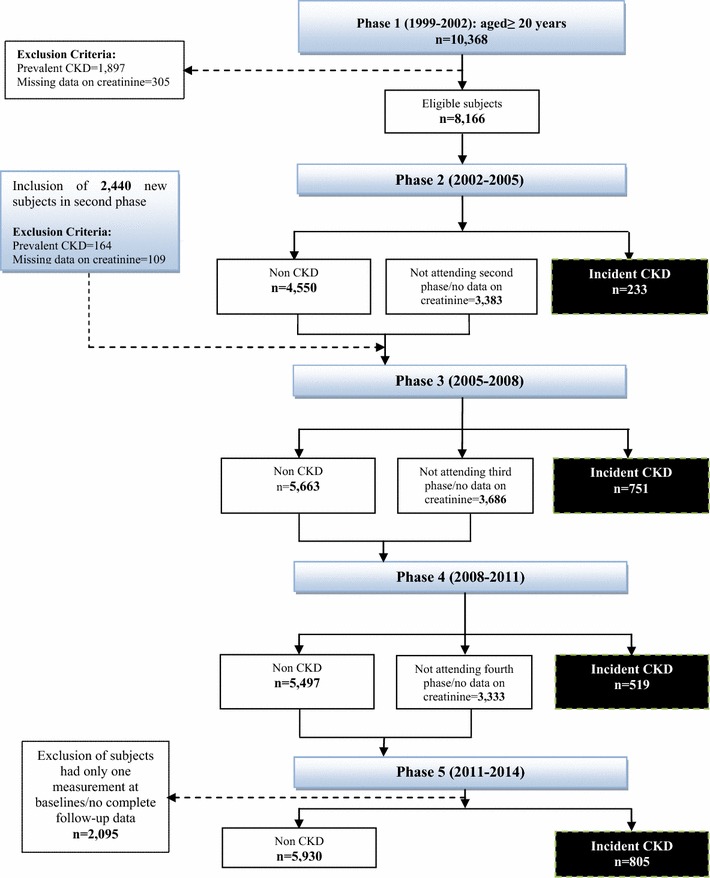
Flow chart for the selection of study subjects, Tehran Lipid and Glucose Study (1999–2014). *CKD* Chronic kidney disease. At first phase of study (1999–2002), 10,368 subjects aged ≥ 20 years participated in the study. After exclusion of 2202 peoples, 8166 eligible subjects were followed in four consecutive phases, i.e. 2002–2005 (phase 2), 2005–2008 (phase 3), 2008–2011 (phase 4) and the final survey in 2011–2014 (phase 5). Another 2440 subjects aged ≥ 20 years were participated in the second phase and after exclusion of 273 subjects, 2167 eligible people were followed in the next 3 phases. Overall, 8238 eligible subjects were followed until 2014, and 2308 new cases of CKD (233 in phase 2; 751 in phase 3; 519 in phase 4 and 805 in phase 5 were identified after a median follow-up of 12.4 years. A total of 2095 subjects lost to follow up due to incomplete follow-up data and 5930 individuals did not developed CKD

The TLGS is an ongoing community-based prospective study performed on a representative sample of residents of Tehran, the capital of the Islamic Republic of Iran. This city composed of 22 urban districts. Study samples were selected from the district 13. The rationales for choosing district 13 were: (1) high stability of the population residing in that district compared to other districts of Tehran, and (2) the age distribution of the population of district 13 was representative of the overall population of Tehran. At first phase of study (1999–2002), a multistage stratified cluster random sampling technique was used to select more than 15,000 people aged ≥ 3 year. The participants then were followed in four consecutive phases, i.e. 2002–2005 (phase 2), 2005–2008 (phase 3), 2008–2011 (phase 4) and the final survey in 2011–2014 (phase 5). Another 3551 residents were invited for the second phase and were followed in the next 3 phases. The details of the study have been published elsewhere [20]. The present study was a post hoc analysis of the TLGS. A total of 12,808 adult participants aged ≥ 20 year from the first and second phases were selected as baseline population and the cohort was re-examined at next phases until the phase 5. Among this participants, we excluded subjects with prevalent CKD (n = 2061) at baseline, and those with missing data on creatinine level (n = 414). Finally, after excluding those without any follow-up data after recruitment (n = 2095), 8238 participants (3795 men and 4443 women) (80% of eligible subjects) were followed until the end of study (Fig. 1). This study was approved by the ethical committee of the Research Institute for Endocrine Sciences of Shahid Beheshti University of Medical Sciences, Tehran, Iran, and conducted in accordance with the Declaration of Helsinki. All participants signed informed consent forms.

### Clinical, anthropometric and laboratory measurements

At baseline, information on demographics, education, smoking status, medical and drug history was collected using interview. Anthropometric measures including body weight and height, hip, wrist and waist circumferences (WC) were measured according to a standard protocol. Body mass index (BMI) was calculated as weight in kilogram divided by the square of height in meter. Blood pressure was measured twice and mean of two measurements was considered as the participant’s blood pressure. Blood samples were drawn after an overnight fast of ≥ 12 h to assess the following parameters: fasting plasma glucose (FPG) (measured by the enzymatic colorimetric glucose oxidase); total cholesterol (TC) and triglycerides (TGs) (were assayed using the enzymatic colorimetric method) and the Serum creatinine (cr) level which was assayed by kinetic colorimetric Jaffe using commercial kits (Pars Azmoon Inc., Tehran, Iran) by a Selectra 2 auto analyzer (Vital Scientific, Spankeren, The Netherlands) [20, 21]. The sensitivity of the assay was 0.2 mg/dl. Reference intervals according to manufacturer’s recommendation were 53–97 mmol/l (0.6–1.1 mg/dl) and 80–115 mmol/l (0.9–1.3 mg/dl) in women and men respectively. Both intra-assay and inter-assay CVs were less than 3.1% in both baseline and follow-up phases. Assay performance was monitored after every 25 tests using lyophilized serum controls in normal and abnormal ranges and all samples were assayed when internal quality control met the standard satisfactory criteria [17, 18]. In the first phase, physical activity level (PAL) was assessed by the lipid research clinic questionnaire (LRC) [22], which was validated for this study [23]. LRC is one of the most common questionnaires to assess PAL in large-scale epidemiological studies [24]. It was replaced by the Modifiable Activity Questionnaire (MAQ) from the 2nd phase for obtaining the quantitative measure of PAL. This questionnaire measures all three forms of activities including leisure time, job, and household activities in the past year [25].

### Definition of variables and outcomes

Kidney function was estimated by the estimated glomerular filtration rate (eGFR) that was calculated using the modification of diet in renal disease (MDRD) equation [26]. The definition of incident CKD was an eGFR of < 60 ml/min per 1.73 m^2^ (CKD Stage 3–5) occurring at any time during the follow-up period. Educational status was categorized to 4 levels as illiterate, 1–9 years, 10–12 years and over 12 years of schooling. Marital status was categorized as single, married, and widowed/divorced. Different categories of smoking status were defined according to WHO guidelines [27]. A current smoker was defined as a person who smokes cigarettes or other smoking implements (water-pipes, or pipes) at least once a day or occasionally. Ex-smoker was formerly daily or occasional smoker who currently does not smoke and non-smoker defined as people who never smoked before. A family history of premature cardio-vascular diseases (CVDs) was considered as any experience of fatal or non-fatal myocardial infarction, stroke or sudden cardiac arrest in first-degree relatives, if it occurred before 55 year of age in male relatives (father, brother and son) and before 65 year of age in female relatives (mother, sister and daughter). Family history of diabetes (FHD) was defined as having type 2 diabetes in first-degree relatives. In first phase, physically active participants were identified as those who were participating in a vigorous physical activity at least 3 days per week [22], and in the second phase, it was defined as achieving a minimum of at least 600 MET (metabolic equivalent task)-minutes per week [28].

### Statistical analyses

Descriptive statistics (mean, SD, proportion) were used to describe baseline characteristics. Comparison of baseline characteristics between men vs. women, between participants with and without incident CKD and between followed and non-followed participants was done using student’s *t* test, Chi square and Mann–Whitney test, as appropriate. Incidence density rate of CKD and respective 95% confidence interval (CI) were calculated for each gender, by dividing the number of events to person-years at risk. Time to event was defined as time of censoring or date of incidence of CKD, whichever occurred first. The event date for the incident cases of CKD was defined as midtime between the date of follow-up visit at which the CKD was diagnosed for the first time, and the most recent follow-up visit prior to the diagnosis. For censored subjects, the time was the interval between the first and the last observation dates. Study participants were censored due to death, loss to follow-up, or the end of the observation period. The Stepwise Cox PH regression model using Akaike information criteria (AIC), as model selection approach, was used to identify significant predictors of incident CKD by calculating multivariable hazard ratios (HRs) with 95% CI (after confirming the proportionality in the model). Due to significant effect modification of gender on FPG, SBP, diastolic blood pressure (DBP), eGFR and WC (all p < 0.05), we stratified our analysis by gender. Moreover, for examination of the gender effect in the multivariable model we fitted the Cox PH model on whole population. In further analysis, conditional survival tree was implemented with the same outcome and covariates as for Cox method [29]. In step 1, among ***m*** covariate, survival tree algorithm selects the variable ***X***_***i***_ with the highest ability to separate survivors and non-survivors using p-values from permutation distributions. In step 2, in order to split *X*_*i*_ into two disjoint sets (dichotomizing), the algorithm first sorts the values of *X*_*i*_ in increasing order. Typically, the midpoint between each pair of adjacent values is considered as a possible cut-point. Therefore, given v values of *X*_*i*_, then v-1 possible splits are evaluated. For each possible cut-point for *X*_*i*_, the value of the log-rank statistic associated with the considered cut-point is computed; then, the cut-point associated with the smallest p-value is selected. This procedure is applied recursively until the tree grew to an optimal number of terminal nodes. A survival tree can naturally group subjects according to their survival time and based on their covariates; hence, it automatically detects complex interactions between covariates without the need to specify them beforehand. In the present study, all baseline variables were entered for constructing survival trees. The minimum criterion for node split was defined as p < 0.05 and the minimum final nodes size were defined as 100 persons. Kaplan–Meier (KM) curves were constructed for each subgroup identified by the survival tree. Also, according the number of subgroups were found by survival tree, we specified one categorical variables with *k* level (*k* is the number of terminal nodes or subgroups in survival trees) and estimated the HR of CKD events among the identified subgroups by considering the low risk group (highest survival probability) as the reference category. We used two packages of Party and Survival from the R software (http://www.r-project.org/) for the analysis. Two-tailed p-values < 0.05 were considered statistically significant.

## Results

### Study participants and baseline characteristics

The study sample consisted of 3795 men, aged 20–84 year (mean, 41.4 ± 14.1) and 4443 women, aged 20–83 year (mean, 37.5 ± 12.1). Table 1 shows the baseline characteristics of the participants. There was a gender difference in all baseline variables except for TC, family history of premature CVD in male relatives, PAL and use of blood glucose lowering drugs. Women were younger and had lower eGFR, WC, wrist circumference, FPG, SBP and DBP, but had higher BMI, hip circumference and heart rate, compared with men (Table 1).

**Table 1:** Baseline characteristics of the study subjects in the TLGS cohort, Tehran Lipid and Glucose Study (1999–2014)

Variables	Total n = 8238	Gender difference		p-value
		Men n = 3795	Women n = 4443	
Age (years)	39.3 (13.3)	41.4 (14.2)	37.5 (12.1)	0.001
Total length of stay in the city (years)	32.9 (13.1)	34.6 (13.2)	31.5 (12.8)	0.001
BMI (kg/m^2^)	26.5 (4.7)	25.8 (4.1)	27.1 (5.1)	0.001
Waist circumference (cm)	87.4 (12.3)	89.1 (11.5)	86.1 (12.8)	0.001
Wrist circumference (cm)	16.7 (1.3)	17.7 (0.9)	15.9 (1.1)	0.001
Hip circumference (cm)	100.29 (9.17)	96.71 (7.23)	103.43 (9.54)	0.001
FPG (mmol/l)	4.9 (4.6–5.4)	5.1 (4.7–5.4)	4.9 (4.5–5.3)	0.001
TG (mmol/l)	1.5 (1.1–2.3)	1.7 (1.2–2.5)	1.4 (0.9–2.1)	0.001
TC (mmol/l)	5.2 (1.1)	5.1 (1.1)	5.2 (1.2)	0.07
HDL-C (mmol/l)	1.1 (0.3)	0.9 (0.2)	1.2 (0.3)	0.001
eGFR (ml/min/1.73 m^2^)	75.6 (10.5)	77.1 (10.8)	74.3 (10.1)	0.001
SBP (mmHg)	116.5 (17.1)	118.8 (16.7)	114.6 (17.1)	0.001
DBP (mmHg)	76.4 (10.6)	77.1(10.7)	75.8 (10.5)	0.001
Heart rate (beats/min)	79.3 (11.5)	75.3 (9.8)	82.7 (11.7)	0.001
Education				
Level 1 (illiterate)	1754 (21.3)	739 (19.5)	1015 (22.8)	
Level 2 (< 9 years)	4831 (58.6)	2228 (58.7)	2603 (58.6)	0.001
Level 3 (9–12 years)	1203 (14.6)	688 (18.1)	515 (11.6)	
Level 4 (> 12 years)	450 (5.5)	140 (3.7)	310 (7.0)	
Marital status				
Single	1337 (16.2)	743 (19.6)	594 (13.4)	
Married	6625 (80.4)	3027 (79.8)	3598 (81.0)	0.001
Divorced/widowed	276 (3.4)	25 (0.7)	251 (5.6)	
FH-CVD in female relatives	669 (8.1)	267 (7.0)	402 (9.0)	0.001
FH-CVD in male relatives	657 (8.0)	310 (8.2)	347 (7.8)	0.568
FHD in first-degree relatives	2255 (27.4)	977 (25.7)	1278 (28.8)	0.002
PAL				
Inactive^a^	5458 (66.3)	2533 (66.7)	2925 (65.8)	0.802
Smoking				
Never	6207 (75.3)	2067 (54.5)	4140 (93.2)	
Past	567 (6.9)	510 (13.4)	57 (1.3)	0.001
Current	1339 (16.3)	1158 (30.5)	181 (4.1)	
Use of blood lipid lowering drugs	177 (2.1)	63 (1.7)	114 (2.6)	0.005
Use of blood glucose lowering drugs	234 (2.8)	100 (2.6)	134 (3.0)	0.319
Use of anti hypertensive drugs	335 (4.1)	113 (3.0)	222 (5.0)	0.001
Use of aspirin	717 (8.7)	394 (10.4)	323 (7.3)	0.001
Use of diuretics	93 (1.1)	22 (0.6)	71 (1.6)	0.001
Prevalence CVD	219 (2.7)	140 (3.7)	79 (1.8)	0.001

Figures are either mean (SD) or N (%) for continuously and categorically distributed variables, respectively

*TLGS* Tehran Lipid and Glucose Study, *BMI* body mass index, *FPG* fasting plasma glucose, *TG* triglyceride, *TC* total cholesterol, *HDL-C* HDL cholesterol, *eGFR* estimated glomerular filtration rate, *SBP* systolic blood pressure, *DBP* diastolic blood pressure, *FH-CVD* family history of premature cardiovascular disease, *FHD* family history of diabetes mellitus, *PAL* physical activity level, *CVD* cardiovascular disease

^a^Doing exercise or labor less than three times a week or performing activities achieving < 600 metabolic equivalent of task (MET)

A comparison of baseline characteristics between followed and non-followed subjects is shown in Table 2. Followed subjects had a significantly higher BMI (26.5 vs. 26.1 kg/m^2^), WC (87.4 vs. 86.3 cm), hip circumference (100.2 vs. 99.6 cm) and TC (5.2 vs. 5.1 mmol/l), but lower FPG (5.2 vs. 5.3 mmol/l) and eGFR (75.5 vs. 76.1) compared to non-followed subjects, respectively.

**Table 2:** Baseline characteristics of followed and non-followed subjects in the TLGS cohort, Tehran Lipid and Glucose Study (1999–2014)

Variables	Total n = 10,333	Followed n = 8238	Non-followed n = 2095	Difference (95% CI)
Age (years)	39.24 (13.64)	39.29 (13.25)	39.02 (15.07)	0.27 (− 0.38, 0.92)
Total length of stay in the city (years)	32.68 (13.47)	32.88 (13.08)	31.88 (14.90)	1.00 (0.30, 1.69)
BMI (kg/m^2^)	26.44 (4.77)	26.50 (4.67)	26.20 (5.14)	0.29 (0.04, 0.54)
Waist circumference (cm)	87.23 (12.38)	87.42 (12.28)	86.47 (12.75)	0.95 (0.33, 1.56)
Wrist circumference (cm)	16.69 (1.34)	16.71 (1.34)	16.64 (1.32)	0.07 (0.01, 0.13)
Hip circumference (cm)	100.18 (9.34)	100.28 (9.17)	99.78 (9.98)	0.50 (0.02, 0.98)
FPG (mmol/l)	5.29 (1.59)	5.27 (1.53)	5.38 (1.83)	− 0.10 (− 0.19, − 0.01)
TG (mmol/l)	1.83 (1.33)	1.84 (1.34)	1.80 (1.33)	0.04 (− 0.02, 0.11)
TC (mmol/l)	5.16 (1.15)	5.17 (1.14)	5.11 (1.17)	0.05 (0.01, 0.11)
HDL-C (mmol/l)	1.07 (0.28)	1.07 (0.28)	1.07 (0.28)	− 0.00 (− 0.01, 0.01)
eGFR (mL/min/1.73 m^2^)	75.70 (10.53)	75.59 (10.49)	76.15 (10.70)	− 0.56 (− 1.07, − 0.04)
SBP (mmHg)	116.59 (17.40)	116.49 (17.05)	116.99 (18.74)	− 0.50 (− 1.33, 0.34)
DBP (mmHg)	76.34 (10.65)	76.39 (10.60)	76.15 (10.81)	0.23 (− 0.27, 0.75)
Heart rate (beats/min)	79.28 (11.54)	79.25 (11.50)	79.40 (11.71)	− 0.15 (− 0.71, 0.40)
Education^a^				
Level 1 (illiterate)	2202 (21.3)	1754 (21.3)	448 (21.4)	0.0086 (− 0.0119, 0.0289)
Level 2 (< 9 years)	6000 (58.1)	4831 (58.6)	1169 (55.8)	
Level 3 (9–12 years)	1520 (14.7)	1203 (14.6)	317 (15.1)	
Level 4 (> 12 years)	611 (5.9)	450 (5.5)	161 (7.7)	
Marital status				
Single	1814 (17.6)	1337 (16.2)	477 (22.8)	0.0759 (0.0540, 0.0979)
Married	8149 (78.9)	6625 (80.4)	1524 (72.7)	
Divorced/widowed	370 (3.6)	276 (3.4)	94 (4.5)	
FH-CVD in female relatives	840 (8.1)	669 (8.1)	171 (8.2)	− 0.0009 (− 0.0293, 0.0275)
FH-CVD in male relatives	843 (8.2)	657 (8.0)	186 (8.9)	− 0.019 (− 0.048, 0.009)
FHD in first-degree relatives	2801 (27.1)	2255 (27.4)	546 (26.1)	0.0107 (− 0.0066, 0.0280)
PAL				
Inactive^b^	6827 (70.5)	5458 (70.7)	1369 (69.9)	− 0.0063 (− 0.0239, 0.0114)
Smoking				
Never	7684 (75.6)	6207 (76.5)	1477 (72.1)	− 0.0001 (− 0.0305, 0.0304)
Past	702 (6.9)	567 (7.0)	135 (6.6)	
Current	1776 (17.5)	1339 (16.5)	437 (21.3)	
Use of blood lipid lowering drugs	231 (2.2)	177 (2.1)	54 (2.6)	− 0.0317 (− 0.0869, 0.0234)
Use of blood glucose lowering drugs	320 (3.1)	234 (2.8)	86 (4.1)	− 0.0681 (− 0.1173, − 0.0189)
Use of anti hypertensive drugs	434 (4.2)	335 (4.1)	99 (4.7)	− 0.0265 (− 0.0667, 0.0138)
Use of aspirin	933 (9.0)	717 (8.7)	216 (10.3)	− 0.0316 (− 0.0599, -0.0034)
Use of diuretics	120 (1.2)	93 (1.1)	27 (1.3)	− 0.0225 (− 0.0976, 0.0526)
Prevalence CVD	324 (3.1)	219 (2.7)	105 (5.0)	− 0.1253 (− 0.1768, -0.0737)

Figures are either mean (SD) or n (%) for continuously and categorically distributed variables, respectively

*TLGS* Tehran Lipid and Glucose Study, *BMI* body mass index, *FPG* fasting plasma glucose, *TG* triglyceride, *TC* total cholesterol, *HDL-C* HDL cholesterol, *eGFR* estimated glomerular filtration rate, *SBP* systolic blood pressure, *DBP* diastolic blood pressure, *FH-CVD* family history of premature cardiovascular disease, *FHD* family history of diabetes mellitus, *PAL* physical activity level, *CVD* cardiovascular disease

^a^The adjusted risk differences (95% CI) between followed and non-followed participants regarding categorical variables were estimated by logistic regression adjrr command in stata

^b^Doing exercise or labor less than three times a week or performing activities achieving < 600 metabolic equivalent of task (MET)

Table 3 shows baseline characteristics of the study participants with and without incident CKD. In both genders, the participants who developed incident CKD were older, had higher BMI, FPG, TG, TC, SBP, DBP, WC and wrist circumferences but lower heart rate and were more likely to be married compared with participants free of CKD. Moreover, frequency of taking lipid, blood glucose and blood pressure lowering drugs as well as aspirin was higher in incident cases of CKD compared to non CKD participants (p < 0.01 for all of these measures).

**Table 3:** Baseline characteristics of the study participants with and without incident CKD, Tehran Lipid and Glucose Study (1999–2014)

Variables	Men			Women		
	Non CKD n = 3002	Incident CKD n = 793	p-value	Non CKD n = 2928	Incident CKD n = 1515	p-value
Age (years)	39.1 (13.3)	50.3 (13.6)	< 0.001	34.2 (10.6)	43.8 (12.4)	< 0.001
Total length of stay in the city (years)	33.1 (12.5)	40.3 (14.1)	< 0.001	28.9 (11.7)	36.5 (13.59)	< 0.001
BMI (kg/m^2^)	25.7 (4.2)	26.3 (3.9)	< 0.001	26.6 (5.1)	28.1 (4.8)	< 0.001
Waist circumference (cm)	88.5 (11.5)	90.9 (11.1)	< 0.001	84.5 (12.7)	88.9 (12.5)	< 0.001
Wrist circumference (cm)	17.6 (0.9)	17.8 (0.9)	< 0.001	15.7 (1.1)	16.1 (1.1)	< 0.001
Hip circumference (cm)	96.6 (7.3)	97.1 (7.1)	0.086	102.8 (9.5)	104.6 (9.4)	< 0.001
FPG (mmol/l)	4.9 (4.7–5.5)	5.2 (4.8–5.7)	< 0.001	4.8 (4.5–5.2)	4.9 (4.7–5.5)	< 0.001
TG (mmol/l)	1.7(1.1–2.5)	1.8(1.3–2.6)	< 0.001	1.3(0.9–1.9)	1.6 (1.1–2.3)	< 0.001
TC (mmol/l)	5.1 (1.1)	5.3 (1.1)	< 0.001	5.1 (1.1)	5.5 (1.3)	< 0.001
HDL-C (mmol/l)	0.9 (0.2)	0.9 (0.2)	0.48	1.1 (0.3)	1.2 (0.3)	0.210
eGFR (mL/min/1.73 m^2^)	79.2(10.6)	69.2 (7.4)	< 0.001	77.1 (10.1)	68.8 (7.1)	< 0.001
SBP (mmHg)	117.1 (15.4)	125.2 (19.7)	< 0.001	111.8 (14.9)	120.1 (19.6)	< 0.001
DBP (mmHg)	76.3 (10.3)	80.1(11.6)	< 0.001	74.6 (10.1)	78.2 (10.8)	< 0.001
Heart rate (beats/min)	75.5 (9.8)	74.47 (10.1)	0.015	83.5(11.8)	81.1 (11.4)	< 0.001
Education						
Level 1 (illiterate)	518 (17.3)	221 (27.9)		560 (19.1)	455 (30.0)	
Level 2 (< 9 years)	1858 (61.9)	370 (46.7)	< 0.001	1884 (64.3)	719 (47.5)	< 0.001
Level 3 (9–12 years)	528 (17.6)	160 (20.2)		361 (12.3)	154 (10.2)	
Level 4 (> 12 years)	98 (3.3)	42 (5.3)		123 (4.2)	187 (12.3)	
Marital status						
Single	686 (22.9)	57 (7.2)		503 (17.2)	91 (6.0)	
Married	2302 (76.7)	725 (91.4)	< 0.001	2325 (79.4)	1273 (84.0)	< 0.001
Divorced/widowed	14 (0.5)	11 (1.4)		100 (3.4)	151 (10.0)	
FH-CVD in female relatives	200 (6.7)	67 (8.4)	0.080	244 (8.3)	158 (10.4)	0.021
FH-CVD in male relatives	256 (8.5)	54 (6.8)	0.116	213 (7.3)	134 (8.8)	0.064
FHD in first-degree relatives	783 (26.1)	194 (24.5)	0.354	822 (28.1)	456 (30.1)	0.157
PAL						
Inactive^a^	2009 (71.2)	524 (69.4)	0.331	1908 (70.4)	1017 (70.9)	0.716
Smoking						
Never	1630 (55.1)	437 (56.2)	0.001	2747 (95.1)	1393 (93.6)	
Past	378 (12.8)	132 (17.0)		33 (1.1)	24 (1.6)	0.101
Current	950 (32.1)	208 (26.8)		109 (3.8)	72 (4.8)	
Use of blood lipid lowering drugs	41 (1.4)	22 (2.8)	0.006	49 (1.7)	65 (4.3)	< 0.001
Use of blood glucose lowering drugs	55 (1.8)	45 (5.7)	< 0.001	54 (1.8)	80 (5.3)	< 0.001
Use of anti hypertensive drugs	59 (2.0)	54 (6.8)	< 0.001	90 (3.1)	132 (8.7)	< 0.001
Use of aspirin	286 (9.5)	108 (13.6)	0.001	172 (5.9)	151 (10.0)	< 0.001
Use of diuretics	12 (0.4)	10 (1.3)	0.004	37 (1.3)	34 (2.2)	0.013
Prevalence CVD	87 (2.9)	53 (6.7)	< 0.001	30 (1.0)	49 (3.2)	< 0.001

Figures are either mean (SD) or N (%) for continuously and categorically distributed variables, respectively

*TLGS* Tehran Lipid and Glucose Study, *BMI* body mass index, *FPG* fasting plasma glucose, *TG* triglyceride, *TC* total cholesterol, *HDL-C* HDL cholesterol, *eGFR* estimated glomerular filtration rate, *SBP* systolic blood pressure, *DBP* diastolic blood pressure, *FH-CVD* family history of premature cardiovascular disease, *FHD* family history of diabetes mellitus, *PAL* physical activity level, *CVD* cardiovascular disease

^a^Doing exercise or labor less than three times a week or performing activities achieving < 600 metabolic equivalent of task (MET)

**Fig. 2:**
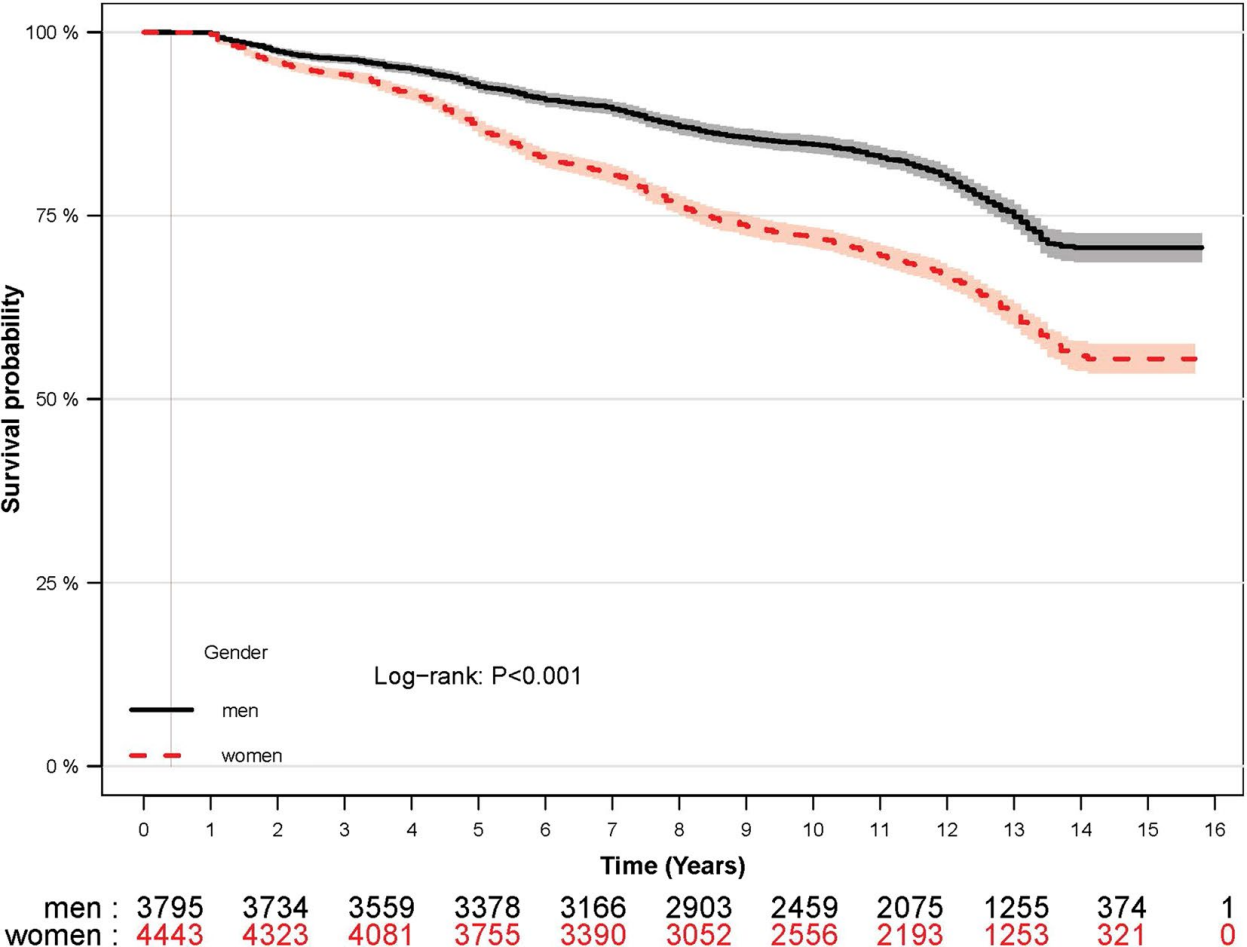
Kaplan–Meier survival curves of male and female subjects are displayed. The number of population at risk in each year of follow up is shown below the plot. The shaded area around the survival curve represents the 95% confidence band. Survival of women (dashed line) was lower than that of men (solid line) during study period (log-rank p < 0.001)

Overall, 2308 new cases of CKD (men = 793 and women = 1515) were identified after a median follow-up of 12.4 [interquartile range (IQR), 9.8–13.6] years. Among incident cases of CKD, 2294 subjects developed stage 3 CKD (eGFR 30–59 ml/min/1.73 m^2^), 12 cases developed stage 4 CKD (eGFR 15–29 ml/min/1.73 m^2^), and 2 cases developed stage 5 CKD (eGFR < 15 ml/min/1.73 m^2^). The incidence density rates of CKD were 20.2 (95% CI 18.8–21.7) and 35.2 (33.5–37.1) per 1000 person-years in men and women, respectively. Figure 2 depicts the survival curve for men and women. Survival of women was lower than that of men during study period (Log-Rank χ^2^ 176, p < 0.001). From the 8238 baseline population, 269 (men = 196) cases of mortality were documented during the study period; of these, 119 (men = 93) cases died from CVD.

### Predictors of incident CKD in men according to Cox model

Among men, age, FPG and DBP were positively associated with the risk of CKD (Table 4). In contrast, WC and TC were negatively associated with the risk of CKD. Also, an inverse association between eGFR and CKD was found in men; every 1-unit increase in eGFR was associated with a 10% reduction in the risk of CKD (HR 0.90; 95% CI 0.89–0.91). The HR for CKD among men with higher education (> 12 years) was 1.56 (1.06–2.28) compared to illiterate group. Being divorced/widower increased the risk of CKD by 204% (1.09–3.84) compared with being married. Interestingly, active men had 21% (1.03–1.42) higher risk of CKD compared to the inactive group. Current smoking was associated with a 24% (1.03–1.48) increase in the risk of CKD. The use of blood glucose lowering drugs at study entry was associated with 59% (1.08–2.35) higher risk of CKD.

**Table 4:** Multivariate Cox regression analysis of factors associated with CKD incidence by gender, Tehran Lipid and Glucose Study (1999–2014)

Variables	HRs	95% Confidence interval	p-value
Men			
Age (years)	1.04	1.03–1.05	< 0.001
eGFR (ml/min/1.73 m^2^)	0.90	0.89–0.91	< 0.001
FPG (mmol/l)	1.10	1.05–1.16	< 0.001
DBP (mmHg)	1.02	1.01–1.03	< 0.001
TC (mmol/l)	0.89	0.83–0.96	0.0025
Waist circumference (cm)	0.98	0.97–0.99	0.016
Hip circumference (cm)	1.02	1.00–1.03	0.075
Heart rate (beats/min)	0.99	0.99–1.00	0.093
Education			
Level 1 (illiterate)	Reference		
Level 2 (< 9 years)	1.18	0.83–1.67	0.34
Level 3 (9–12 years)	1.24	0.87–1.78	0.23
Level 4 (> 12 years)	1.56	1.06–2.28	0.021
Marital status			
Married	Reference		
Single	1.06	0.77–1.47	0.70
Divorced/widowed	2.04	1.09–3.84	0.025
FH–CVD in female relatives			
No	Reference		
Yes	1.25	0.97–1.62	0.080
PAL			
Inactive^a^	Reference		
Active	1.21	1.03–1.42	0.016
Smoking			
Never	Reference		
Past	1.06	0.87–1.30	0.51
Current	1.24	1.03–1.48	0.017
Use of blood glucose lowering drugs			
No	Reference		
Yes	1.59	1.08–2.35	0.017
Use of aspirin			
No	Reference		
Yes	0.84	0.67–1.04	0.116
Women			
Age (years)	1.02	1.02–1.03	< 0.001
eGFR (mL/min/10.73 m^2^)	0.91	0.91–0.92	< 0.001
FPG (mmol/L)	1.05	1.02–1.08	< 0.001
Heart rate (beats/min)	0.99	0.98–0.99	< 0.001
Waist circumference (cm)	0.99	0.98–0.99	0.002
SBP (mmHg)	1.01	1.00–1.01	0.028
Wrist circumference (cm)	1.05	1.00–1.01	0.119
FHD in first-degree relatives			
No	Reference		
Yes	0.90	0.79–1.01	0.08
Prevalence CVD			
No	Reference		
Yes	1.35	0.99–1.82	0.048

The Stepwise Cox PH regression model using AIC as model selection approach was used to identify significant predictors of incident CKD by calculating multivariable hazard ratios (HRs) with 95% CI

*TLGS* Tehran Lipid and Glucose Study, *PH* proportional hazard, *AIC* Akaike information criteria, *TLGS* Tehran Lipid and Glucose Study, *FPG* fasting plasma glucose, *TC* total cholesterol, *eGFR* estimated glomerular filtration rate, *SBP* systolic blood pressure, *DBP* diastolic blood pressure, *FH-CVD* family history of premature cardiovascular disease, *FHD* family history of diabetes mellitus, *PAL* physical activity level, *CVD* cardiovascular disease

^a^Doing exercise or labor less than three times a week or performing activities achieving < 600 metabolic equivalent of task (MET)

### Predictors of incident CKD in women according to Cox model

The associations of age, FPG and WC with incidence of CKD in women were similar to those observed for men (Table 4). Every 1-unit increase in eGFR was associated with a 9% reduction in the risk of CKD (HR 0.91; 0.91–0.92). Additionally, SBP and heart rate were significantly associated with CKD incidence. Prevalent CVD at baseline was associated with a 35% (0.99–1.82) increase in the risk of CKD.

### Predictors of incident CKD in whole population according to Cox model

In the whole population, women had more than double risk (HR 2.09; 1.82–2.39) of CKD compared with men. Moreover, age, FPG, and DBP were positively associated with CKD incidence. In contrast, eGFR, WC, TC and heart rate were negatively associated with the risk of CKD. Every 1-unit increase in eGFR was associated with a 9% reduction in the risk of CKD (HR 0.91; 0.90–0.92). Prevalent CVD at baseline was associated with a 25% (1.01–1.54) increase in the risk of CKD. The HR for CKD among individuals with higher education (> 12 years) was 1.41 (1.14–1.72) compared to illiterate individuals (Table 5).

**Table 5:** Multivariate Cox regression analysis of factors associated with CKD incidence in total population, Tehran Lipid and Glucose Study (1999–2014)

Variables	HR	95% CI	p-value
Gender			
Male	Reference		
Female	2.09	1.82–2.39	< 0.001
Age (years)	1.03	1.02–1.03	< 0.001
eGFR (ml/min/1.73 m^2^)	0.91	0.90–0.92	< 0.001
Waist circumference (cm)	0.99	0.98–0.99	< 0.001
FPG (mmol/l)	1.06	1.03–1.09	< 0.001
Heart rate (beats/min)	0.99	0.98–0.99	< 0.001
TC (mmol/l)	0.95	0.91–0.99	0.043
DBP (mmHg)	1.01	1.00–1.01	0.045
SBP (mmHg)	1.00	0.99–1.00	0.056
Wrist circumference (cm)	1.05	0.99–1.10	0.077
Education			
Level 1 (illiterate)	Reference		
Level 2 (< 9 years)	1.05	0.89–1.24	0.541
Level 3 (9–12 years)	1.11	0.92–1.33	0.255
Level 4 (> 12 years)	1.41	1.14–1.72	0.001
Marital status			
Married	Reference		
Single	0.90	0.74–1.09	0.312
Divorced/widowed	1.19	0.99–1.42	0.057
FHD in first-degree relatives			
No	Reference		
Yes	0.90	0.82–0.99	0.045
Use of blood glucose lowering drugs			
No	Reference		
Yes	1.22	0.96–1.56	0.097
PAL			
Inactive^a^	Reference		
Active	1.09	0.99–1.20	0.064
Prevalence CVD			
No	Reference		
Yes	1.25	1.01–1.54	0.035

The Stepwise Cox PH regression model using AIC as model selection approach was used to identify significant predictors of incident CKD by calculating multivariable hazard ratios (HRs) with 95% CI

*TLGS* Tehran Lipid and Glucose Study, *PH* proportional hazard, *CI* confidence interval, *AIC* Akaike information criteria, *FPG* fasting plasma glucose, *TC* total cholesterol, *eGFR* estimated glomerular filtration rate, *SBP* systolic blood pressure, *DBP* diastolic blood pressure, *FHD* family history of diabetes mellitus, *PAL* physical activity level, *CVD* cardiovascular disease

^a^Doing exercise or labor less than three times a week or performing activities achieving < 600 metabolic equivalent of task (MET)

### Survival tree in men

**Fig. 3:**
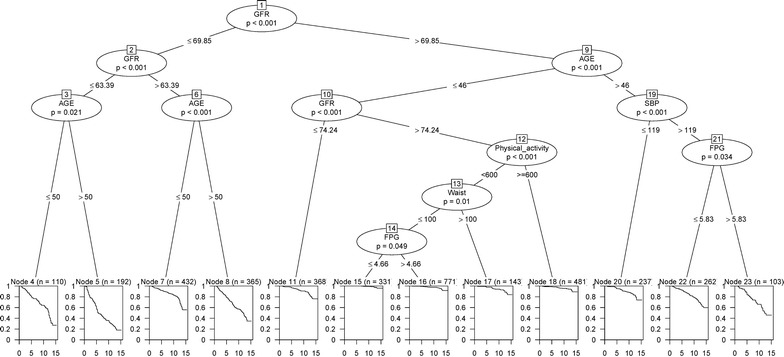
Survival tree for incidence of CKD events in men. Squares represent terminal nodes; numbers (n) in squares denote sample size (top line), and curves inside the squares show the Kaplan–Meier estimated survival of subpopulations. Circles represent the most significant variable based on log-rank (LR) and permutation P for splitting population to smaller groups. Six variables were entered as the most important predictors of the occurrence of CKD over a 12.4 year period. Node 1 at the top of figure shows that eGFR is the most important variable for first split (p < 0.001). The best cutoff value for eGFR was 69.85 ml/min/1.73 m^2^; accordingly, male populations were divided into two groups: left (≤ 69.85) and right (> 69.85) branches. This procedure was applied recursively until the tree grew to an optimal number of terminal nodes. Therefore, 12 groups were identified by the survival tree algorithm. Each path from the first node to a terminal node specifies a combination of predictors and their cutoff values leading to a terminal node forms an interaction pattern. Each interaction pattern specifies a subgroup of individuals with similar survival probability. For example, node 4 shows the survival probability for a group of men with eGFR between 63.39 and 69.85 and aged ≤ 50 years which is worse than nodes 15 and 16. *SBP* systolic blood pressure, *FPG* fasting plasma glucose, eGFR estimated glomerular filtration rate

**Fig. 4:**
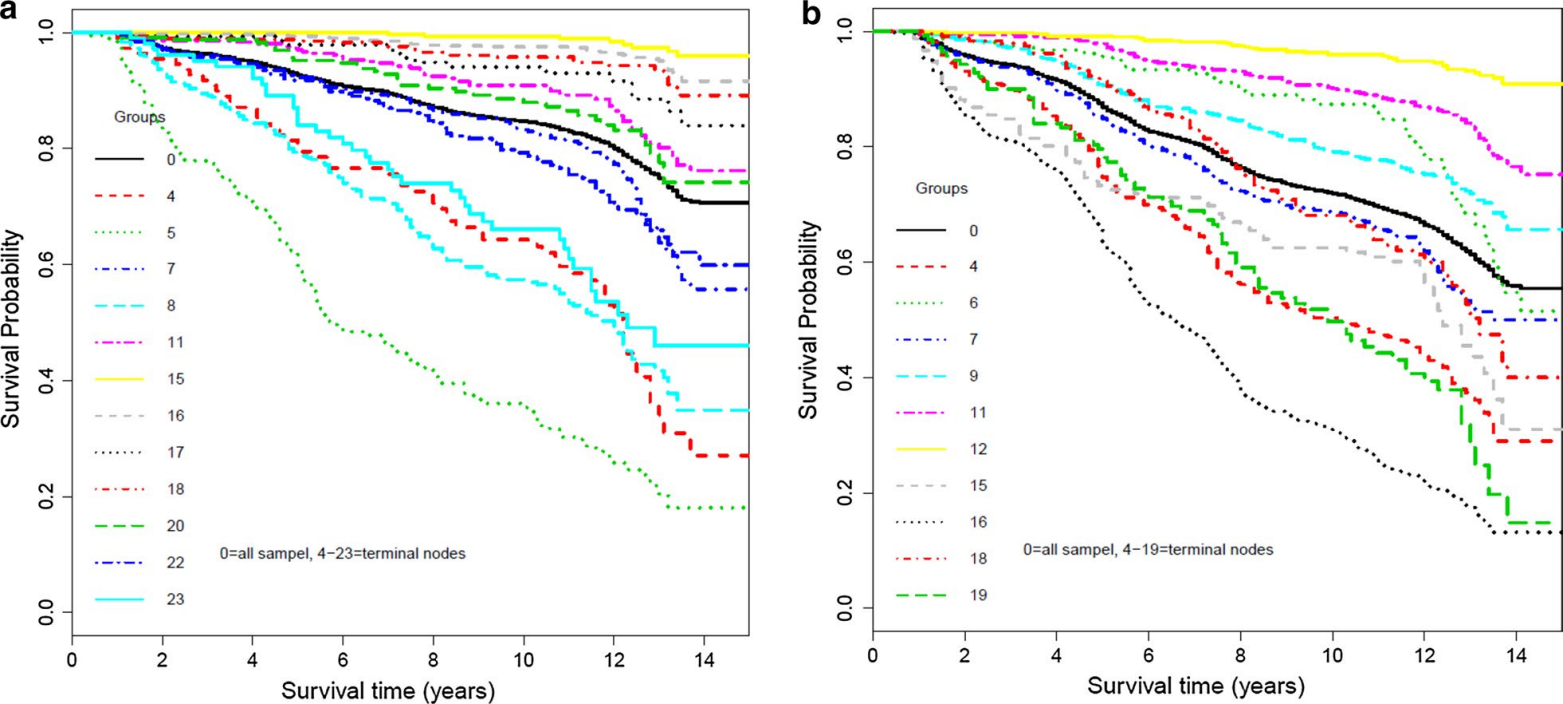
The Kaplan–Meier survival curves of the terminal nodes identified by the survival tree analysis. The numbers on the plots display the nodes number. The black solid line shows survival curve of total population, and other lines show the survival curve of each terminal node. **a** Kaplan–Meier survival of 12 nodes of the survival tree of Fig. 3 for male population. It can be seen that the curves of nodes 4, 5, 7, 8, 22 and 23 are lower than the whole population’s curve, showing that they cover high risk groups for the CKD events. The symmetric remark can be made for nodes (11, 15, 16, 17, 18 and 20) which have a survival probability above the whole population’s curve which cover low risk groups for the CKD events among men. **b** Kaplan–Meier survival of 10 nodes of the survival tree of Fig. 5 for female population. The survival curves of nodes 16, 4, 19, 15, and 7 are lower than the whole population’s curve, and survival curves of nodes 12, 11, 6, and 9 have a survival probability above the whole population’s curve, showing the high risk and low risk groups for the CKD events among women, respectively

The results of the survival tree in men are presented in Fig. 3. The eGFR, age, FPG, PAL and WC were selected by the tree to group the male population. The best cutoff values for these predictors were found by the survival tree algorithm. The eGFR was found as the most important predictor for CKD with a cutoff value of 69.8 ml/min/1.73 m^2^. The group with eGFR ≤ 69.8 ml/min/1.73 m^2^ (left side of the tree) was further split by age; so that, the group with eGFR < 63.4 ml/min/1.73 m^2^ and age > 50 year had the highest risk for CKD. The lowest risk was found among younger men aged ≤ 46 year and eGFR > 74 ml/min/1.73 m^2^. A moderate risk was explored among the groups with eGFR > 69.85 ml/min/1.73 m^2^ and age > 46 year; survival probability among these groups depended on the SBP and FPG. So that, the group with SBP > 119 mmHg and FPG > 5.8 mmol/l had the lowest survival. Ultimately, 12 groups were identified by the survival tree algorithm. The Kaplan–Meier curves for these groups are shown in Fig. 4a. The curves of nodes 4, 5, 7, 8, 22 and 23 were lower than the whole population’s curve, showing that they covered high risk groups for the CKD events among men. The symmetric remark can be made for nodes (11, 15, 16, 17, 18 and 20) which had a survival probability above the whole population’s curve which covered low risk groups for the CKD events in men. Using node 15 with the lowest risk as the reference group, HRs of CKD was estimated for all other groups (nodes). Accordingly, node 5 had the highest risk for CKD incidence, compared to node 15 (HR 70.6; 34.6–144.5; p < 0.001) (Table 6).

**Table 6:** Cox analyses of patterns identified using survival tree in the male population, Tehran Lipid and Glucose Study (1999–2014)

Nodes	Number of cases/number of events	Pattern description	HRs (95% CI)	p-value
15	331/8	eGFR > 74 ml/min/1.73 m^2^, age ≤ 46 year, PAL < 600 MET, WC ≤ 100 cm, FPG ≤ 4.7 mmol/l	Reference	–
4	110/58	eGFR ≤ 63.4 ml/min/1.73 m^2^, age ≤ 50 year	34.67 (16.54–72.65)	< 0.001
5	192/130	eGFR ≤ 63.4 ml/min/1.73 m^2^, age > 50 year	70.68 (34.57–144.52)	< 0.001
7	432/130	63.4 < eGFR ≤ 74 ml/min/1.73 m^2^, age ≤ 50 year	14.91 (7.30–30.45)	< 0.001
8	365/172	63.4 < eGFR ≤ 74 ml/min/1.73 m^2^, age > 50 year	35.82 (17.61–72.85)	< 0.001
11	368/62	70 < eGFR ≤ 74 ml/min/1.73 m^2^, age ≤ 46 year	7.48 (3.58–15.62)	< 0.001
16	771/39	eGFR > 74 ml/min/1.73 m^2^, age ≤ 46 year, PAL < 600 MET, WC ≤ 100 cm, FPG > 4.7 mmol/l	2.16 (1.01–4.63)	< 0.05
17	143/14	eGFR > 74 ml/min/1.73 m^2^, age ≤ 46 year, PAL < 600 MET, WC > 100 cm	4.67 (1.95–11.13)	< 0.001
18	481/27	eGFR > 74 ml/min/1.73 m^2^, age ≤ 46 year, PAL ≥ 600 MET	3.02 (1.37–6.67)	< 0.001
20	237/37	eGFR > 70 ml/min/1.73 m^2^, age > 46 year, SBP ≤ 119 mmHg	8.63 (4.02–18.55)	< 0.001
22	262/75	eGFR > 70 ml/min/1.73 m^2^, age > 46 year, SBP > 119 mmHg, FPG ≤ 5.8 mmol/l	15.47 (7.46–32.08)	< 0.001
23	103/41	eGFR > 70 ml/min/1.73 m^2^, age > 46 year, SBP > 119 mmHg, FPG > 5.8 mmol/l	27.26 (12.77–58.18)	< 0.001

Column 1 shows the number of terminal nodes of survival tree of Fig. 3 for male population. Column 2 displays the sample size and number of events in each terminal node. Column 3 represents the patterns of each terminal node. Column 4 shows the HR for each node compared to node with high survival probability as the reference node

*CI* Confidence intervals, *HRs* Hazard ratio, *SBP* Systolic blood pressure, *eGFR* Estimated glomerular filtration rate, *FPG* Fasting plasma glucose, *PAL* Physical activity level, *WC* Waist circumference, *MET* Metabolic equivalent of task

### Survival tree in women

**Fig. 5:**
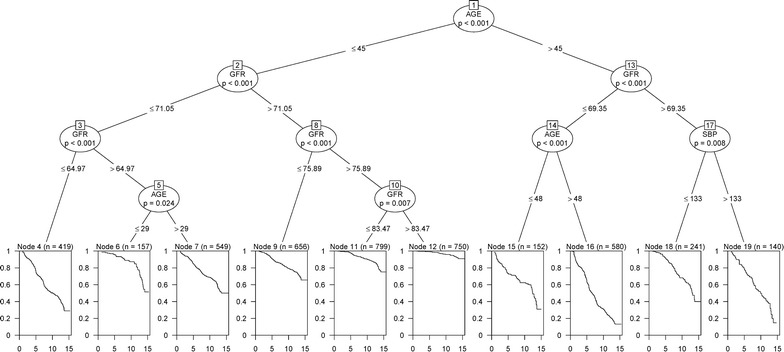
Survival tree for incidence of CKD events in women. Squares represent terminal nodes; numbers (n) in squares denote sample size (top line), and curves inside the squares show the Kaplan–Meier estimated survival of subpopulations. Circles represent the most significant variable based on log-rank (LR) and permutation P for splitting population to smaller groups. Three variables were entered as the most important predictors of the occurrence of CKD over a 12.4 year period. Node 1 at the top of figure shows that age is the most important variable for first split (p < 0.001). The best cutoff value for age was 45 years; accordingly, female populations were divided into two groups: left (≤ 45 year) and right (> 45 year) branches. This procedure was applied recursively until the tree grew to an optimal number of terminal nodes. Therefore, 10 groups were identified by the survival tree algorithm. Each path from the first node to a terminal node specifies a combination of predictors and their cutoff values leading to a terminal node forms an interaction pattern. Each interaction pattern specifies a subgroup of individuals with similar survival probability. For example, node 4 shows the survival probability for a group of women aged ≤ 45 year with eGFR of ≤ 64.97 mL/min/*1.73* m^2^ which is worse than survival of nodes 12. *SBP* systolic blood pressure, *eGFR* estimated glomerular filtration rate

The survival tree for women is shown in Fig. 5. Three variables (age, eGFR and SBP) were found as the most significant predictors associated with CKD. Initially, the participants were split by age with the cutoff value of 45 year (p < 0.001). On the right side of the tree, the group aged > 45 year was split by eGFR with a cutoff value of 69.35 ml/min/1.73 m^2^; then, the group with eGFR > 69.35 ml/min/1.73 m^2^ was split by SBP with a cutoff value of 133 mmHg. Further inspection of the tree showed that in age > 45 year, a SBP > 133 mmHg can significantly increase the risk of CKD. Among women in younger age (< 45 year), those with eGFR > 83.5 ml/min/1.73 m^2^ (node 12) and eGFR < 65 ml/min/1.73 m^2^ (node 4) had the lowest and highest risk, respectively, for incidence of CKD. The Kaplan–Meier curves for the ten groups are shown in Fig. 4b. The survival curves of nodes 16, 4, 19, 15, and 7 were lower than the whole population’s curve, and survival curves of nodes 12, 11, 6, and 9 had a survival probability above the whole population’s curve, showing the high risk and low risk groups for the CKD events among women, respectively. As Table 7 shows, node 16 had the highest risk for CKD incidence, compared to node 12 as the reference group (HR 27.25; 19.88–37.34; p < 0.001).

**Table 7:** Cox analyses of patterns identified using survival tree in the female population, Tehran Lipid and Glucose Study (1999–2014)

Nodes	Number of cases/number of events	Pattern description	HRs (95% CI)	p-value
12	750/43	Age ≤ 45 year, eGFR > 83.5 ml/min/1.73 m^2^	Reference	–
4	419/247	Age ≤ 45 year, eGFR ≤ 65 ml/min/1.73 m^2^	15.18 (10.97–20.99)	< 0.001
6	157/41	Age ≤ 29 year, 65 ≤ eGFR ≤ 71 ml/min/1.73 m^2^	5.07 (3.30–7.77)	< 0.001
7	549/220	29 ≤ Age ≤ 45 year, 65 ≤ eGFR ≤ 71 ml/min/1.73 m^2^	8.78 (6.32–12.15)	< 0.001
9	656/166	Age ≤ 45 year, 71 ≤ eGFR ≤ 76 ml/min/1.73 m^2^	4.93 (3.52–6.90)	< 0.001
11	799/124	Age ≤ 45 year, 75 ≤ eGFR ≤ 83.5 ml/min/1.73 m^2^	2.72 (1.92–3.86)	< 0.001
15	152/74	45 ≤ Age < 48 year, eGFR ≤ 69 ml/min/1.73 m^2^	12.06 (8.28–17.57)	< 0.001
16	580/423	Age > 48 year, eGFR ≤ 69 ml/min/1.73 m^2^	27.25 (19.88–37.34)	< 0.001
18	241/97	Age > 45 year, eGFR > 69 ml/min/1.73 m^2^, SBP ≤ 133 mmHg	8.95 (6.25–12.82)	< 0.001
19	140/80	Age > 45 year, eGFR > 69 ml/min/1.73 m^2^, SBP > 133 mmHg	16.41(11.32–23.80)	< 0.001

Column 1 shows the number of terminal nodes of survival tree of Fig. 5 for female population. Column 2 displays the sample size and number of events in each terminal node. Column 3 represents the patterns of each terminal node. Column 4 shows the HR for each node compared to reference node with high survival probability

*CI* Confidence intervals, *HRs* Hazard ratio, *SBP* Systolic blood pressure, *eGFR* Estimated glomerular filtration rate

## Discussion

The results of the present study showed that more than 20 and 35% of men and of women, respectively, developed CKD during 15 years follow-up. Results of the multivariate Cox PH models showed that women had more than double risk for CKD incidence compared to men. The eGFR, age, FPG and WC were significant predictors for incident CKD in both genders and whole population. However, in men, DBP, TC, current smoking, being divorced/widower, higher educational levels (> 12 year), taking blood glucose lowering drugs and PAL were significantly associated with an increased risk of CKD. In women, it was found that SBP, heart rate and prevalent CVD were significantly associated with CKD in addition to the four common predictors.

Compared to other population-based studies in the US, Europe and Japan [30], the incidence rate of CKD was higher in our study, despite the lower mean age of the population. The higher incidence of CKD in our study population could be due to life style changes, leading to a sharp rise in risk factors of chronic diseases [31]. In line with our previous study [32], we found female gender and aging as an independent factor associated with CKD, as reported by other studies [30, 33].

Interestingly, Cox models showed that higher WC was associated with lower risks of CKD among both genders. Some studies have shown that a higher WC was associated with the lower risk of CKD [32]; while a number of studies reported no significant association [34]. Surprisingly, we found a negative association between TC and incident CKD in men and whole population. The association between CKD and dyslipidemia has not been demonstrated in prospective studies, and is still subject to controversy [35, 36].

Hypertension is recognized as a strong predictor for progression of renal dysfunction [32]. We found a positive and significant relationship between hypertension and CKD in both genders and whole population. In line with other studies [37], we showed a positive association between FPG and incidence of CKD in both genders and whole population. In the present study, among whole population and men, individuals with high educational levels (> 12 years of education), showed increased risk for CKD compared to illiterates. In our previous study, a similar association was found in women [32]. However, inverse association has been reported in other cross sectional and prospective studies [38]. The positive association between high educational levels and incident CKD observed in our study may be attributed to the direct and indirect impact of higher education on higher income. Since high income is almost coincides with high fat, protein and salt consumption, it affects the incident CKD through increasing prevalence of some chronic disease such as hypertension, diabetes, and obesity [39]. Further investigations are needed in order to better clarify this relationships.

Smoking is known as an independent risk factor for progression to CKD [37, 40]. Our study also showed that current smokers had increased risk of CKD compared to never smokers only in men.

To date, studies assessing the association between physical activity and CKD incidence have provided conflicting results [41]. Surprisingly, we found 21% higher risk of CKD in “active” men.

We found significant association between elevated heart rate and lower risks of CKD incidence among women and whole population. This association is contrary to the results of Brotman [42], who reported an association between higher resting heart rate and CKD incidence. As heart rate is affected by many interior and exterior factors, more studies are needed to explain this unexpected result.

Compared with the general population, patients with CKD have an unacceptably higher risk for premature death, primarily as a result of CVD [43]. In our study, prevalent CVD at baseline was associated with risk of developing CKD among women and whole population.

### Interaction patterns identified by the survival trees

According to survival tree analysis, eGFR and age were found as the most important predictors for CKD incidence among men and women, respectively. Current evidences from cross sectional and longitudinal studies suggest that decline in GFR is a universal phenomenon which happens after age 30 in both men and women, and the rate of decline accelerates after age 50–60 years [44]. This decline appears to be a part of the normal physiologic process and is associated with structural changes in the kidney [45].

Survival tree in woman (Fig. 5) shows that not only aging is the most important predictor for CKD progression, but suggesting that the critical threshold of age for developing CKD is much lower than 30. As Fig. 5 shows, a group of women with age ≤ 29 year and eGFR between 65 and 71 ml/min/1.73 m^2^ had a moderate risk of developing CKD during the follow-up period (node 6).

According to our findings, the lowest risk was found among women aged ≤ 45 year and with eGFR > 83 ml/min/1.73 m^2^; suggesting that the threshold of ≥ 83 ml/min/1.73 m^2^ for eGFR is a safe value for decreasing the risk of CKD among women aged ≤ 45 year.

Figure 5 also shows that among women aged > 45 year, a SBP > 133 mmHg (high-range pre-hypertension and hypertension) was significantly associated with increased risk of CKD even with eGFR > 69 ml/min/1.73 m^2^. According to a recent study, the overall prevalence of prehypertension was approximately 46% among adult Iranian population [46]; therefore, the effective strategies for the prevention and treatment of prehypertension is required to be considered among Iranian women.

Overall, the survival tree in women found 10 prognostic groups, defined only by the age, eGFR and SBP; it does not mean that other variables have no predictive ability in the CKD incidence. If the tree is growing in full depth, other variables may be involved in the prediction of CKD; however, the number of subjects in the terminal nodes will be so small for significant results.

Survival tree in male population identified 12 subgroups with different patterns (Fig. 3). The highest risk was found among men with eGFR < 63 ml/min/1.73 m^2^ (nodes 4 and 5).

As we previously mentioned, the Cox PH model found negative association between WC and PAL in relation to risk of CKD among male population (Table 4). These unexpected results may be explained by the interactions found by the survival tree; as the Fig. 3 shows, low PAL and WC > 100 cm was significantly associated with increased risk of CKD (node 17); while, WC < 100 cm and FPG ≤ 4.7 mmol/l improved survival rate among inactive men (node 15). These findings suggest that there is a particularly strong interaction between these factors in relation to CKD incidence among male population. Survival tree also found a group of men aged > 46 year with a SBP > 119 mmHg (prehypertension and hypertension) and FPG > 5.83 mmol/l (pre-diabetes and diabetes), who had moderate risk of CKD incidence. In line with our findings, Yamagata et al. [37] showed that hypertension and diabetes were independent predictor for developing CKD. Because of the high prevalence and incidence of pre-hypertension, hypertension, pre-diabetes and diabetes among Iranian population [47], more attention should be paid to early intervention and risk factor modification for this high risk group.

In summary, survival trees are the helpful exploratory tools that have been applied in recent years in different medical studies [48–51]. However, investigators should be aware of their drawbacks and limitations. A major issue with survival trees is that they can be built to fit the training data too perfectly (over fit); while it may not perform well on other unknown data [52]. We used the conditional inference survival tree developed by Hothorn et al. [53], which is shown to be more reliable and less likely to over fit compared to the other survival tree algorithms.

This study is subject to some limitations. First, our study was a post hoc analysis of a prospective cohort that was not able to control for some covariates related to CKD such as family history of kidney disease that is related to decline in kidney function beginning at early ages [54]. Additionally, no quantitative urinary indexes were available at the baseline or follow-up visit, because, the cohort was not originally designed for kidney diseases. Second, this study has been conducted on a sample of Iranian population and further studies should be conducted to determine whether our findings could be applicable to other populations. Third, as inherent to any prospective cohort study, the level of risk factors at the baseline examination might change during the follow up, e.g. some degree of misclassification may have occurred in this study, leading to biased estimated hazard ratios towards the null. Fourth, about 20% of participants were lost to follow-up in our study; we found statistically but not clinically important differences between the followed vs. non-followed population in some baseline variables. In total population, mean values of FPG and eGFR were lower, and TC and WC were higher in followed compared to non-followed population. Since FPG and eGFR were reported to be the most important predictors for CKD among Iranian population [32], we may underestimate the incidence of CKD in our study. To evaluate the extent to which lost to follow-up may have influenced measures of association in final models, we calculated propensity scores in both genders and in the total population. The PS is the conditional probability that an individual participate in the follow-up, given a set of covariates [55]. To obtain PS, we applied multivariable logistic regression with participation in the follow-up as the outcome and all baseline variables as the predictors. The estimated probability of participation, or the PS, derived from the multivariable logistic regression model described above was added to the final Cox PH models. In general, in both genders and total population, there were no changes in estimated HRs and CIs for predictors in the Cox PH models. We also added PS as a covariate to develop survival tree models along with all baseline covariates; interestingly, adding the PS did not alter the structure of survival tree in both genders and total population (data not shown). Therefore, lost to follow-up did not cause selection bias in our study. And last but not least, our diagnosis of CKD was based on a single estimate of GFR, which we acknowledge tends to overestimate the incidence of kidney disease [37].

As strengths, this is the first study that identified interactions between CKD risk factors in a well-characterized cohort of adults using the survival tree approach. We used data from a large, long-term, population-based cohort of men and women. In fact, in men, the statistical power of our study to detect a HR of 20, 30 and 50% for incident CKD was 72, 95 and 99%; the corresponding values in women were 94, 99 and 100%, respectively. We assessed the actual measurement of blood pressure and laboratory parameters, rather than self-reported data. In addition, we investigated a wide range of variables as potential predictors.

As, there will never be enough resources to implement every programme for all population, health policy makers prefer interventions that target high-risk groups [56]. Our study, identified not only risk factors of CKD in total population by Cox model but also identified high-risk groups by survival tree analysis, which can be used for implement specific interventions for each group according their level of risk.

## Conclusions

Cox regression models is suitable for demonstrating the relation of a predictor to the outcome; while, survival trees are ideally suited for exploration of some prognostic groups of individuals by detecting certain types of interactions without the need to specify them beforehand. In our study, Cox PH models identified the main effects of the several traditional risk factors of CKD; however, survival tree analysis identified a number of interactions between those risk factors. Survival trees created 12 subgroups with different patterns of eGFR, age, FPG, physical activity level and WC in men. Among women, age, eGFR and SBP interacted with each other and identified 10 subgroups. In conclusion, these two statistical methods can be deemed to be somewhat complementary for survival data analysis. The prognostic groups could potentially be valuable for early detection and management of high risk individuals for CKD in the public health setting and at clinic.

## Abbreviations

AIC: Akaike information criteria
BMI: body mass index
CI: confidence interval
CKD: chronic kidney disease
CVD: cardiovascular disease
Cox PH: Cox proportional hazards
DBP: diastolic blood pressure
eGFR: estimated glomerular filtration rate
ESRD: end stage renal disease
FHD: family history of diabetes
FPG: fasting plasma glucose
HR: hazard ratios
IQR: inter quartile range
KM: Kaplan–Meier
MET: metabolic equivalent of task
MDRD: modification of diet in the renal disease study
PAL: physical activity level
PH: proportional hazards
PS: propensity score
SBP: systolic blood pressure
TC: total cholesterol
TGs: triglycerides
TLGS: Tehran Lipid and Glucose Study
WC: waist circumference
